# Novel LncRNA ZFHX4-AS1 as a Potential Prognostic Biomarker That Affects the Immune Microenvironment in Ovarian Cancer

**DOI:** 10.3389/fonc.2022.945518

**Published:** 2022-07-12

**Authors:** Xiaoyan Wang, Yiwen Wang, Fusheng Sun, Yang Xu, Zhaocong Zhang, Chang Yang, Lijie Zhang, Ge Lou

**Affiliations:** ^1^ Department of Gynecology, Harbin Medical University Cancer Hospital, Harbin, China; ^2^ Department of Surgery, The First Affiliated Hospital of Harbin Medical University, Harbin, China

**Keywords:** lncRNA ZFHX4-AS1, ovarian cancer, prognosis, biomarker, tumor-infiltrating immune cells, immune microenvironment, immunoinhibitors, immune checkpoints

## Abstract

**Background:**

Ovarian cancer (OvCa) is a malignant disease of the female reproductive system with a high mortality rate. LncRNA has been confirmed to play a crucial role in the development and progression of various cancer types. Novel lncRNA ZFHX4-AS1 has been reported in several cancers, albeit its functional mechanisms in OvCa remain unclear.

**Methods:**

With reference to the public databases and based on integrating bioinformatics analyses, we explored the expression of ZFHX4-AS1 and its roles in the prognosis of OvCa. We employed the Kaplan-Meier curves to investigate the outcome of patients with different ZFHX4-AS1 expressions. Furthermore, its biological function and the related hallmark pathways were assessed through Gene Ontology (GO) annotation, Kyoto Encyclopedia of Genes and Genomes (KEGG) pathway analyses, and Gene-set enrichment analysis (GSEA). We explored the correlation between lncRNA ZFHX4-AS1 and tumor-infiltrating immune cells through CIBERSORT. The immune checkpoints associated with lncRNA ZFHX4-AS1 and its related genes were investigated. The effect of lncRNA ZFHX4-AS1 on proliferation, invasion and migration of OvCa cells was verified through Cell Counting Kit (CCK)-8, colony formation, wound healing and transwell assays.

**Results:**

The expression of lncRNA ZFHX4-AS1 was upregulated in OvCa relative to that in normal tissues. Increased lncRNA ZFHX4-AS1 expression was associated with poor overall survival and progression-free survival in OvCa. The GO and KEGG pathway analyses revealed the role of lncRNA ZFHX4-AS1 in cell metabolism, protein synthesis, cell proliferation, and cell cycle. GSEA indicated the hallmark gene sets that were significantly enriched in the high and low expression groups. The CIBERSORT database revealed M2 macrophages, memory B-cells, naïve B cells, and activated NK cells were affected by lncRNA ZFHX4-AS1 expression (all *P* < 0.05). The expression of lncRNA ZFHX4-AS1 and its related differential genes *MRPS11*, *NSA2*, and *MRPL13* were significantly correlated with the immune checkpoints. Knockdown of lncRNA ZFHX4-AS1 could inhibit the proliferation, invasion and migration of OvCa cells.

**Conclusions:**

The results suggested that lncRNA ZFHX4-AS1 is a novel prognostic biomarker associated with cell proliferation, metabolism, infiltration, and distribution of tumor-infiltrating immune cells in OvCa, indicating that lncRNA ZFHX4-AS1 can be used as a potential therapeutic target for OvCa in the future.

## Introduction

Among gynecological cancers, ovarian cancer (OvCa) has the highest mortality rate, with an average five-year survival rate of only about 47% (1). Owing to the lack of obvious early clinical symptoms and specific detection and diagnosis methods, the majority of the patients are diagnosed at an advanced stage, leading to a poor prognosis and a high recurrence rate (2). Presently, the auxiliary diagnosis of OvCa is achieved by imaging examination combined with certain biomarkers such as CA125. The UK Collaborative Trial of Ovarian Cancer Screening revealed that annual multimodal screening based on serum CA125 or annual transvaginal ultrasound screening did not effectively reduce the mortality rate over a median follow-up of 16.3 years (3). Hence, the diagnosis and treatment of OvCa are fraught with challenges. Looking for effective prognostic biomarkers for the diagnosis and treatment of OvCa and investigating the tumorigenesis and progression mechanisms has become the current research trend.

Long non-coding RNAs (lncRNAs) are a set of non-protein-coding RNAs that are > 200 nucleotides in length, and they are mainly transcribed by RNA polymerase II (4). LncRNAs have been proven to be involved in the regulation of many intracellular processes, especially chromatin dynamics regulation, gene expression, growth, differentiation, and development (5). A variety of lncRNAs are abnormally expressed in different disease types, and some of them are closely correlated with tumorigenesis and development (6–9). To date, many pieces of research have shown that some lncRNAs, such as HOTAIR, H19, and MALAT1, are involved in many detrimental biological processes, including the development and progression of malignant tumors. The lncRNAs also serve as molecular biomarkers indicating poor prognosis of the patients (10–12). In the current study, the differentially expressed lncRNAs in OvCa tissues and normal tissues were firstly screened out from The Cancer Genome Atlas (TCGA) database and Genotype-Tissue Expression (GTEx) database, and then the lncRNAs that have a significant impact on the survival of OvCa patients were analyzed. We selected the annotated lncRNA ZFHX4-AS1, which is highly expressed in ovarian cancer. LncRNA ZFHX4-AS1 is a newly identified lncRNA that is located on 8q21.13. In previous research, lncRNA ZFHX4-AS1 has been confirmed to be highly expressed in bladder cancer (13). Moreover, it has been proven to affect cell invasion and migration by regulating the FAT4-dependent Hippo signaling pathway in breast cancer (14). However, its role in OvCa remains unclear.

In this research, the expression level of lncRNA ZFHX4-AS1 was investigated and it was presented as a negative prognostic biomarker in OvCa by making use of publicly available databases. The proprietary genomic variation and functional mechanism related to the expression of lncRNA ZFHX4-AS1 were explored. Furthermore, the fractions of tumor-infiltrating immune cells associated with lncRNA ZFHX4-AS1 in the tumor microenvironment of OvCa and the correlation between lncRNA ZFHX4-AS1 and the related differential genes and immunoinhibitors were explored. Our findings suggested that lncRNA ZFHX4-AS1 could be a potential treatment target or a prognostic biomarker and that it could be associated with immune cell infiltration and immune checkpoints in OvCa.

## Methods

### Collection of Data and Preprocessing

In our study, the Fragments per Kilobase of Transcript per Million Mapped Reads (FPKM) for the RNA-Seq data of OvCa tissues from TCGA database and the normal tissues from GTEx database were downloaded from the University of California Santa Cruz Xena (UCSC Xena; https://xena.ucsc.edu/) platform. The latest clinical data of TCGA was downloaded from the Genomic Data Commons (GDC), and patients without the clinical information were excluded. Then, we converted the FPKM data into the Transcripts Per Million (TPM)reads for our analyses. Survival analysis of the data excluded samples without RNA sequencing data and patients with overall survival (OS) time of < 30 days. The validation expression data was sourced from the Gene Expression Omnibus (GEO) datasets and the International Cancer Genome Consortium (ICGC) datasets; the GEO datasets included GSE26193 and GSE18520.

### Kaplan–Meier Plotter Analysis

The prognostic value of lncRNA ZFHX4-AS1 in pan-cancer was analyzed by Kaplan-Meier plotting. The OS of the high and low expression groups was assessed and analyzed by hazard ratio (HR), 95% confidence interval (CI), and logrank *P* value.

### Differential Expression Genes Analyses

Through comparison of the high and low expression groups of lncRNA ZFHX4-AS1, DEGs were identified by using the limma R package, and the threshold was set to |log 2-fold change (FC)|> 1.5, with *P*<0.05.

### Construction and Evaluation of Nomogram

The predictive model of nomograms to forecast the prognosis of OvCa patients was established according to the multivariate Cox analysis. On the basis of the prognostic model, the risk score of the clinicopathological features and the lncRNA expression level for each patient were calculated as the total score to forecast the prognosis of OvCa patients. The precision of the nomogram prediction was estimated according to the calibration diagram. The statistical tests were two-tailed, and the level of statistical significance was set to 0.05.

### Enrichment Analysis

The Gene Ontology (GO) and Kyoto Encyclopedia of Genes and Genomes (KEGG) pathway enrichment analyses were conducted for DEGs in the high and low lncRNA ZFHX4-AS1 expression groups by using the DAVID database (DAVID; http://www.david.niaid.nih.gov).

### Gene Set Enrichment Analysis

GSEA was performed by using the clusterprofiler package on DEGs sorted from large to small by logFC. We selected the cancer hallmarks with a false discovery rate (FDR) of < 0.07 and *P* < 0.05 considered to indicate statistical significance. The gene sets adopted in this article were h.all.v7.2.symbols.gmt, which were downloaded from the Molecular Signatures Database (MSigDB; http://software.broadinstitute.org/gsea/msigdb/index.jsp).

### Immune Cell Infiltrations Analysis

Immunoinvasion of 22 tumor-infiltrating immune cells in the OvCa tumor samples was quantified by using the cibersort R package. The specific names of the 22 immune cells are listed in **Figure 7E**. Spearman correlation was applied to analyze the correlation between lncRNA ZFHX4-AS1 and the distribution of the 22 immune cells. Wilcoxon rank sum test was performed on immune cells that infiltrated differentially between the high and low lncRNA expression groups.

### Protein–ProteinInteractions Network Construction

As a public online database, the Search Tool for the Retrieval of Interacting Genes (STRING; https://string-db.org/) was adopted to predict the functional interactions among proteins. We considered an interaction score of >0.4 as the cut-off criteria. We accordingly constructed the hub genes regulatory network from the extraction of the top 10% of genes contained in the PPI network.

### TISIDB Analysis

The TISIDB database (TISIDB; http://cis.hku.hk/TISIDB) integrates 988 reported immune-related anti-tumor genes, para-cancer multi-omics data, containing various immunology data resources obtained from 7 public databases. The relationship between 3 of the DEGs with top 5 degree and immunoinhibitors was analyzed with reference to the TISIDB database.

### Study Subjects

The tissue samples of 12 patients with OvCa were primary surgical resected specimens obtained from the Department of Gynecology, Harbin Medical University Cancer Hospital. We used the relative normal tissue samples from 10 patients as the control. Clinicopathologic characteristics of 12 patients with OvCa were provided in **Supplementary Table 1**. The Ethics Committee of the Harbin Medical University Cancer Hospital approved the study, and all patients provided their signed written consent. All tissue specimens were frozen in liquid nitrogen immediately after excision for total RNA extraction.

### Quantitative Real-Time PCR

According to the manufacturer’s instruction, total RNA from the clinical samples were collected with Trizol reagent (Invitrogen, Carlsbad, USA). We used the lnRcute lncRNA First-Strand cDNA Kit (Tiangen Biotech, Beijing, China) for reverse transcription procedures. The RT-qPCR was cycled with the ABI StepOnePlus Real-Time PCR system using the lnRcute lncRNA qPCR Kit (SYBR Green; Tiangen Biotech). Specific primer sequences for lncRNA ZFHX4-AS1, GAPDH, CD206, PDCD1LG2, and CTLA4 were listed in the **Supplementary Table 2**.

### Cell Culture

OvCa cell lines SKOV-3 and A2780 were purchased from Shanghai Chuanqiu Biotechnology Co., Ltd. (China). A human normal ovarian epithelial cell line(IOSE80) was acquired from the BeNa Culture Collection (China).SKOV-3 and IOSE80 cell lines were cultured with Roswell Park Memorial Institute(RPMI) 1640 medium (Gibco, Beijing ThermoFisher Biochemical Products, Co., Ltd., China) supplemented with 10% fetal bovine serum(FBS), whereas the A2780 cell line was cultured in Dulbecco’s Modified Eagle Medium (Gibco) supplemented with 10% FBS. Each culture was kept in a humid incubator with a 5% CO_2_ atmosphere at 37°C.

### Knockdown Studies

The logarithmic growth-phase cells were selected and seeded into a 6-well plate at the density of 3.5 × 10^5^ cells/well a day before transfection. When the cells were incubated up to 60% density, the transfection was performed as per the instructions of jetPRIME transfection reagent (Polyplus Transfection, Strasbourg, France). Then, SKOV-3 and A2780 cell lines were transfected with siRNA and the negative control to knockdown the lncRNA ZFHX4-AS1 expression. The sequence of siRNA ZFHX4-AS1 is depicted in the **Supplementary Table 3**. The transfection efficiency of each cell line was detected by RT-qPCR assay conducted 24 h after transfection.

### Cell Proliferation Assay

The cell proliferation was detected by using the Cell Counting Kit-8 (CCK-8; TargetMol, Shanghai, China) as per the manufacturer’s instructions after different treatments. Specifically, 2× 10^3^ cells/well were seeded into 96-well plates and cultured at 37°C and under 5% CO_2_ overnight. Next, CCK-8 (10 μL) was added into each well to detect the extent of cell proliferation at 1–5 days by determining the optical density of each well at 450 nm with a microplate reader (BioTek ELx800Winooski, VT, USA).

### Colony Formation Assay

The cells were seeded in 6-well plates with 800 cells per well and cultured for about 12 days. When obvious cell colony formation was visible to the naked eye, the cells were fixed with 1ml 4% paraformaldehyde in each well for half an hour, washed gently with PBS once, and 1ml crystal violet dye was added to each well. After dyeing for half an hour, the excess crystal violet was washed with pure water, and then photographed and counted.

### Wound Healing Assay

After the cells were counted, SKOV-3 cells (6×10^5^ cells per well) and A2780 cells (8×10^5^ cells per well) were seeded into 6-well plates and cultured overnight at 37°C with 5% CO2. The cells were scratched with a straight line using a 200-µl micropipette. After washing the cells with PBS for three times, basal medium was added. The scratch healing was recorded at 0 h, 24 h and 48 h in the same field under a photographic microscope.

### Transwell Migration and Invasion

The cells were re-suspended in serum-free medium.After cell counting, the cell concentration was adjusted to 2×10^5^/mL, and 200µl cell suspension was added to the upper compartment of transwell plates (Corning, NY, USA). In the invasion assay, the upper chamber was covered with a mixture of medium and Matrigel (Corning). We added 800µl medium containing 10% FBS to the lower chamber and the cells were cultured for 24-48 hours. Finally, the lower surface cells were fixed with 4% paraformaldehyde, stained with crystal violet, photographed with a microscope and counted.

### Western Blotting

The cell protein was separated by 10% SDS-polyacrylamide gels electrophoresis and transferred to PVDF membranes. After blocking with 5% skim milk powder for 2 hours, the membrane was incubated with primary antibodies against E-cadherin (1:5000, Proteintech Group, Chicago, IL, USA), vimentin ((1:2000, Proteintech Group), N-cadherin (1:2000, Proteintech Group) and β-actin (1:10000, Proteintech Group) at 4°C overnight. Then the membrane was incubated with rabbit or mouse secondary antibodies at room temperature for 1 hour, and the protein bands were detected using ECL detection system (Tanon-5200Multi, Shanghai, China).

### Statistical Analysis

All bioinformatics statistical analyses and graphs were prepared using the R software (Version 4.0.3). Chi-squared test, Kruskal–Wallis (KW) test, and Wilcoxon rank sum test were adopted to analyze the correlations between the clinicopathological characteristics and the lncRNA ZFHX4-AS1 expression. Kaplan-Meier survival curves with log-rank test were employed to evaluate the OS and progression-free survival (PFS) of OvCa patients in different lncRNA ZFHX4-AS1 expression groups. The receiver operating characteristic (ROC) curve was plotted by using the ROC it software package to evaluate the sensitivity and specificity values of lncRNA ZFHX4-AS1 in OvCa. The results of the univariate and multivariate Cox logistic regression model determined the lncRNA ZFHX4-AS1 expression and the clinicopathological factors affecting the survival time. Experimental data are presented as the mean ± S.D. The GraphPad Prism 8.0 was used for performing statistical analyses and mapping. The comparisons between the two groups were statistically analyzed by Student’s *t-*test. The two-way analysis of variance ANOVA was conducted for multiple comparisons. *P* values in all analyses were two-sided, and *P* < 0.05 was considered to indicate statistical significance.

## Results

### The Expression Level of LncRNA ZFHX4-AS1 in Patients With OvCa

**Figure 1:**
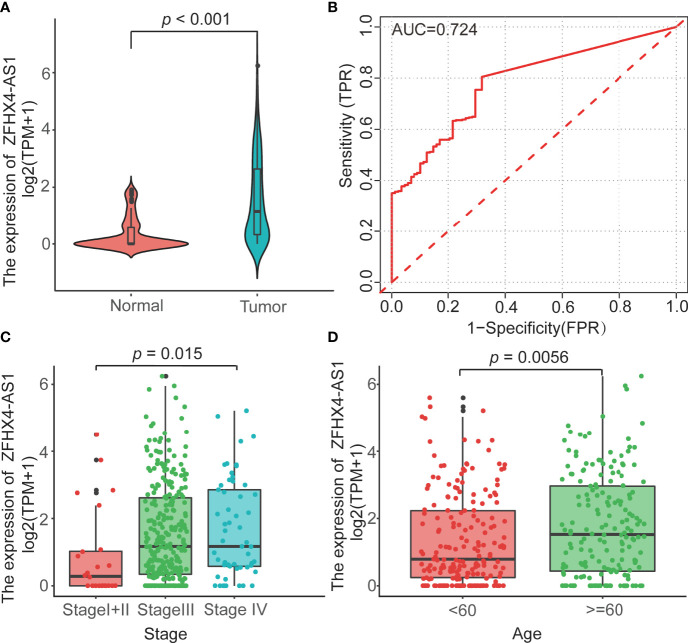
**(A)** The lncRNA ZFHX4-AS1 expression levels of tumorous tissues in TCGA and normal tissues in the GTEx cohorts. *P*<0.001. **(B)** The ROC curves with the lncRNA ZFHX4-AS1 expression in TCGA. **(C, D)** Correlation between the lncRNA ZFHX4-AS1 expression and the clinicopathologic characteristics in OvCa, including the FIGO stage and age.

To investigate the differential expression of lncRNA ZFHX4-AS1 in OvCa, the gene expression and clinical data of 379 patients with OvCa were obtained from TCGA, as shown in **Table 1**. The Wilcoxon rank sum test analysis showed that lncRNA ZFHX4-AS1 had a high expression in 379 OvCa tissues as against 88 normal ovarian tissues (*P* < 0.001) (**Figure 1A**). ROC analysis was performed on data pertaining to the OvCa and normal samples to determine the sensitivity and specificity values of lncRNA ZFHX4-AS1, which resulted in an AUC of 0.724 (**Figure 1B**).

**Table 1:** Correlation between ZFHX4-AS1 expression and clinicopathologic characteristics of OvCa in the validation cohort.

Characters	Level	Low expression of ZFHX4-AS1	High expression of ZFHX4-AS1	*P*
N		105	263	
Stage (%)	Stage I+II	14 (13.3%)	9 (3.5%)	<0.001
	Stage III	81 (77.2%)	207 (79.6%)	
	Stage IV	10 (9.5%)	44 (16.9%)	
Grade (%)	Grade1+2	16 (15.7%)	30 (11.7%)	0.4022
	Grade3	86 (84.3%)	226 (88.3%)	
Lymphatic invasion (%)	Yes	31 (63.3%)	68 (70.1%)	0.5173
	No	18 (36.7%)	29 (29.9%)	
Venous invasion (%)	Yes	25 (61%)	39 (61.9%)	1
	No	16 (39%)	24 (38.1%)	
Race (%)	White	93 (88.6%)	228 (86.7%)	0.7529
	Others	12 (11.4%)	35 (13.3%)	
Age (%)	<60	62 (59.1%)	132 (50.2%)	0.1552
	>=60	43 (40.9%)	131 (49.8%)	

### LncRNA ZFHX4-AS1 Expression Is Correlated With the Clinicopathological Features of OvCa

The TCGA cohort was used to scrutinize the lncRNA ZFHX4-AS1 expression in OvCa and analyze its correlation with the clinicopathological factors. In the study cohort, 368 cases were divided into lncRNA ZFHX4-AS1 high and low expression groups through maximally selected rank statistics. As shown in **Table 1** and **Figures 1C, D**, the higher expression of lncRNA ZFHX4-AS1 was significantly correlated with advanced FIGO stage (*P* = 0.013) and older age (*P* = 0.0056). Univariate logistic regression analysis showed that lncRNA ZFHX4-AS1 expression was associated with poor clinicopathological outcomes in OvCa as a categorical dependent variable. As presented in **Table 2**, Increased expression of lncRNA ZFHX4-AS1 demonstrated a significant correlation with the FIGO stage (odds ratio (OR) = 0.2 for Stage I+II vs. Stage III+IV, *P* = 0.001).

**Table 2:** LncRNA ZFHX4-AS1 expression associated with clinicopathologic characteristics (logistic regression).

Characteristics	Total (N)	Odds Ratio (OR)	*P* value
Stage (I+II vs III+IV)	365	0.2 (0.08-0.53)	0.001
Grade (1 + 2 vs 3)	358	1.11 (0.59-2.11)	0.749
Lymphatic invasion	146	1.12 (0.56-2.27)	0.742
Venous invasion	104	0.78 (0.35-1.74)	0.55
Race (white vs others)	368	0.7 (0.38-1.29)	0.25
Age (>=60 vs <60)	368	1.45 (0.95-2.21)	0.084

Cox model was used to perform univariate analysis of the prognostic factors affecting OS. In univariate analysis, higher lncRNA ZFHX4-AS1expression signified worse OS (*P* = 0.012, hazard ratio (HR) = 1.47[95%CI 1.09–1.98]). Moreover, there was a correlation between older age and poorer OS (*P* = 0.049, HR = 0.77[95%CI 0.59–1.00]) (**Table 3**). Subsequently, multivariate analysis was performed using the Cox regression model, and the results showed that the expression of lncRNA ZFHX4-AS1 was independently associated with OS (*P* = 0.0038, HR = 2.7[95%CI1.38-5.3]), whereas the other prognostic factors were not significantly related to OS (**Table 3**).

**Table 3:** Univariate and multivariate regression survival models for prognostic covariates of OvCa patients.

		Univariate analysis		Multivariate analysis	
	Total (N)	HR (95%CI)	*P* value	HR (95%CI)	*P* value
Stage (I+II vs III+IV)	365	2.04 (0.91-4.61)	0.085	1.08 (0.39 - 2.97)	0.8846
Grade (1 + 2 vs 3)	358	1.2 (0.81-1.78)	0.357	0.91 (0.39 - 2.12)	0.8292
Lymphatic invasion	146	0.75 (0.44-1.27)	0.284	0.64 (0.36 - 1.13)	0.1263
Venous invasion	104	1.12 (0.61-2.06)	0.723	–	–
Race (white vs others)	368	0.68 (0.44-1.03)	0.07	0.58 (0.28 - 1.22)	0.1532
Age (>=60 vs <60)	368	0.77 (0.59-1)	0.049	1.68 (0.95 - 2.97)	0.0736
Group (high vs low)	368	1.47 (1.09-1.98)	0.012	2.7 (1.38 - 5.3)	0.0038

### LncRNA ZFHX4-AS1 Expression Level Predicts Prognosis in Pan-Cancer and OvCa

Kaplan-meier plotter database was used to analyze the effect of lncRNA ZFHX4-AS1 on OS in different kinds of cancers to evaluate its prognostic value. As shown in the **Supplementary Figures 1, 2**, high expression of lncRNA ZFHX4-AS1 predicted shorter OS in several cancers, such as bladder carcinoma, head-neck squamous cell carcinoma, kidney renal clear cell carcinoma, stomach adenocarcinoma, uterine corpus endometrial carcinoma and OvCa, etc.

**Figure 2:**
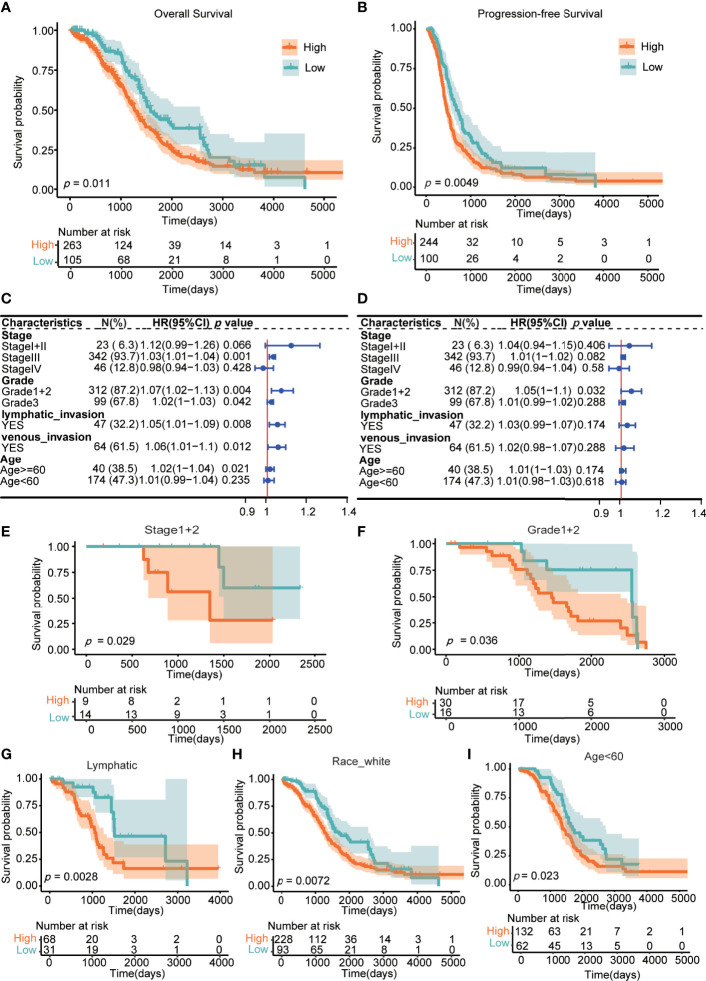
Association between the lncRNA ZFHX4-AS1 expression and prognosis in OvCa. **(A)** for overall survival (OS), **(B)** for progression-free survival (PFS). The forest plot indicated the effect of the expression of lncRNA ZFHX4-AS1 in the presence of clinicopathological characteristics for **(C)** OS, **(D)** PFS. Prognostic values of the differential expression of lncRNA ZFHX4-AS1 in different subgroups, including **(E)** the FIGO stage I+II, **(F)** Grade 1 + 2, **(G)** lymphatic invasion, **(H)** Race-white, **(I)** Age < 60 years.

In the TCGA cohorts, the high and low expression groups of lncRNA ZFHX4-AS1 were compared. According to the Kaplan–Meier survival curves, patients in the low expression group gained a more satisfactory OS than those in the high expression group (*P* = 0.011) (**Figure 2A**). Additionally, increased lncRNA ZFHX4-AS1 expression was distinctly correlated with poor PFS (*P* = 0.0049) (**Figure 2B**). Also, in the case of different clinicopathological characteristics, univariate Cox hazard analysis was performed to determine the influence of lncRNA ZFHX4-AS1 expression on OS and PFS individually (**Figures 2C, D**).

Differentially expressed lncRNA ZFHX4-AS1exhibited prognostic value in some clinicopathological subgroups, including stage I+II, grade1+2, lymphatic invasion, race-white, and age < 60 years (*P* < 0.05) (**Figures 2E–I**). From the above analysis, it is evident that increased lncRNA ZFHX4-AS1 expression predicts a potentially poor prognosis.

### Construction of Nomogram to Investigate and Validate the Function of LncRNA ZFHX4-AS1

**Figure 3:**
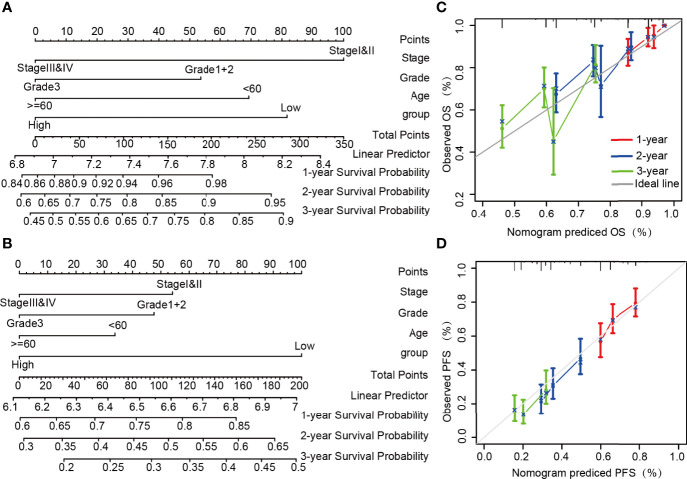
Construction and performance validation of nomogram based on the lncRNA ZFHX4-AS1 expression in OvCa patients. Nomogram to predict **(A)** OS, **(B)** PFS for OvCa patients. The calibration curve and Hosmer–Lemeshow test of nomograms in the TCGA OvCa cohort for **(C)** OS and **(D)** PFS.

In combination with the clinicopathological risk factors, the nomogram was constructed as a clinical prognostic assessment tool for patients with OvCa. The integrated clinicopathological features of the nomogram involved stage, grade, age, and group. Lower total scores in the nomogram for OS and PFS represented a poorer prognosis (**Figures 3A, B**). From the calibration curves of OS and PFS nomograms, it can be inferred that the predicted results agree well with the observed findings in the patients and that there is no deviation from the perfect fit in the testing. As shown in **Figures 3C, D**, the deviation corrected line in the calibration diagram is close to the ideal curve, which denotes that the predicted probability is in stable agreement with the actual observed values. Succinctly, the nomogram can serve as a good predictor of survival in patients with OvCa.

### Functional Analysis and Predictive Signaling Pathways of LncRNA ZFHX4-AS1

**Figure 4:**
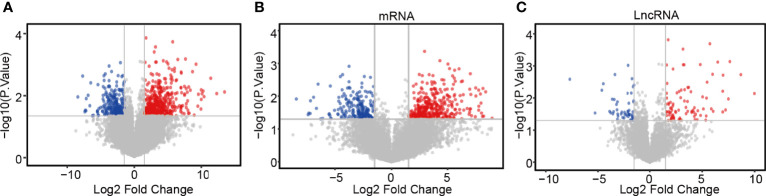
Differential expression gene screening between the high and low expression groups of lncRNA ZFHX4-AS1. **(A)** Volcanic plot of differentially expressed gene profiles between the high and low expression groups of lncRNA ZFHX4-AS1. **(B)** Differential mRNA and **(C)** lncRNA profiles between the lncRNA ZFHX4-AS1 high and low expression groups.

The DEGs and co-regulatory genes associated with the expression of lncRNA ZFHX4-AS1 in OvCa are likely to reveal the underlying mechanisms of ZFHX4-AS1. The limma R package was employed to analyze the data from TCGA, and 769 DEGs (including 497 upregulated and 272 downregulated ones), 139 differentially expressed lncRNAs (including 87 upregulated and 52 downregulated ones), and 630 differentially expressed mRNAs (including 410 up regulated and 220 downregulated ones) were identified (**Figures 4A–C**).

**Figure 5:**
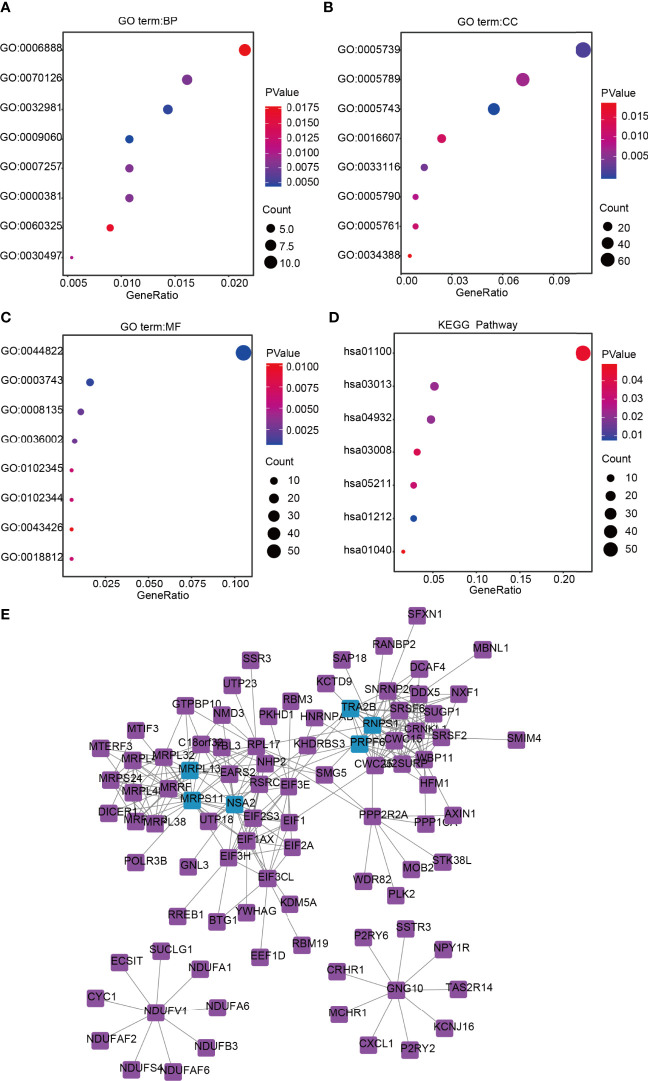
Functional annotation and signaling pathways prediction of lncRNA ZFHX4-AS1 in OvCa. Gene Ontology (GO) was applied and shows the top 8 of **(A)** BP, **(B)** CC, and **(C)** MF. **(D)** The KEGG pathway was analyzed according to the DEGs of lncRNA ZFHX4-AS1, and the TOP 8 pathways were mapped. **(E)** The protein-protein interaction network of lncRNA ZFHX4-AS1-related DEGs.

The DAVID database was used for GO and KEGG analyses of DEGs related to lncRNA ZFHX4-AS1 in OvCa. The top eight enrichment results in three functional groups were further clustered and displayed in the form of bubble plots (**Figures 5A–D**). The biological processes principally consisted of aerobic respiration, mitochondrial respiratory chain complex I assembly, mitochondrial translational termination, regulation of alternative mRNA splicing *via* spliceosome, and activation of JUN kinase activity. The enriched cellular components involved were the mitochondrion, endoplasmic reticulum membrane, mitochondrial ribosome, and nuclear speck. The molecular functions chiefly encompassed translation factor activity, RNA binding, translation initiation factor activity, pre-mRNA binding, and myogenic regulatory factor binding. According to the KEGG enrichment analysis, the DEGs were correlated with RNA transport, ribosome biogenesis in eukaryotes, metabolic pathways, renal cell carcinoma, fatty acid metabolism, and biosynthesis of unsaturated fatty acids. Based on the above analysis, lncRNA ZFHX4-AS1 appears to have a potential influence on cellular aerobic respiration, cell metabolism, and protein synthesis.

Subsequently, based on the analysis in the String database, the protein interactions among the genes related to lncRNA ZFHX4-AS1and their relationships in an interactive network were researched, as shown in **Figure 5E**. The genes with top-five degrees are highlighted in the figure.

### Gene Set Enrichment Analysis of Genes Related to LncRNA ZFHX4-AS1

**Figure 6:**
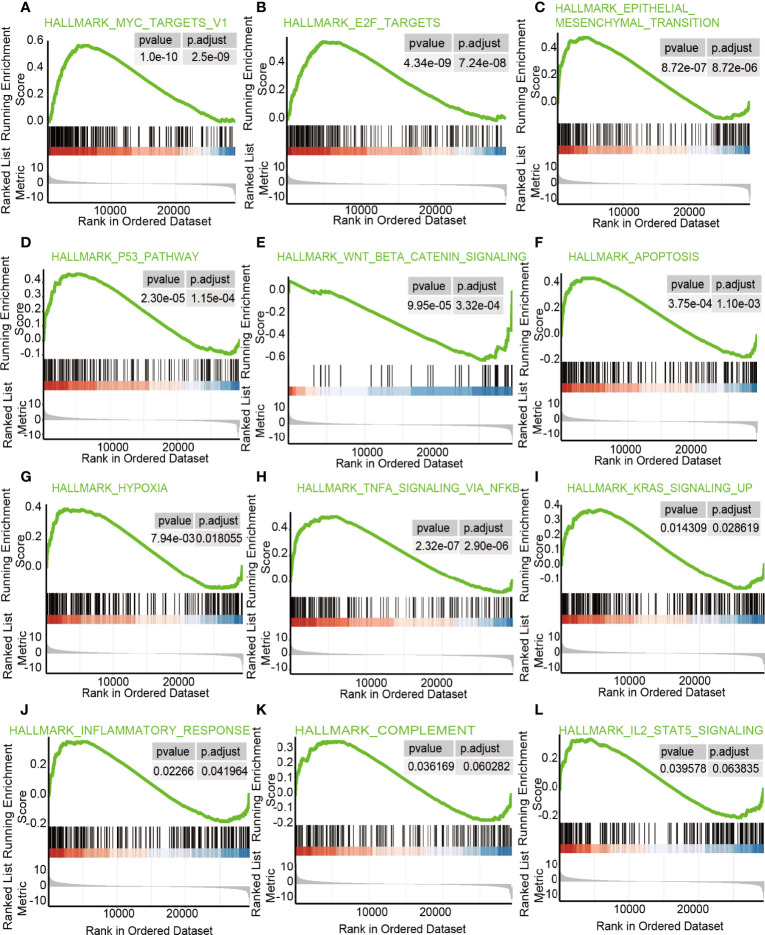
The enrichment hallmarks of OvCa from the GSEA.GSEA results displayed **(A)** MYCtargetsv1 **(B)** E2F targets, **(C)** epithelial mesenchymal transition, **(D)** P53 pathway, **(E)** Wnt/β-catenin signaling, **(F)** apoptosis, **(G)** hypoxia, **(H)** TNFa signaling *via* NF-kB, **(I)** KRAS signaling up, **(J)** inflammatory response, **(K)** complement, and **(L)** IL-2-STAT5 signaling.

GSEA was performed on the groups with differentially expressed lncRNA ZFHX4-AS1 to uncover various signal pathways in OvCa. In the high lncRNA ZFHX4-AS1 expression set, 30 out of the 50 genes were upregulated and significantly enriched when *P* < 0.05, NES > 1.0, and FDRq < 0.07. As shown in **Figure 6**, the enriched hallmark gene sets correlated with immune response were as follows: “TNFASIGNALINGVIANFKB, ” “KRASSIGNALINGUP, ” “INFLAMMATORYRESPONSE, ” “COMPLEMENT, ” and “IL2STAT5SIGNALING.” The enriched hallmark sets of genes involved in tumorigenesis and progression were “MYCTARGETSV1, ” “E2FTARGETS”, “EPITHELIALMESENCHYMALTRANSITION”, “P53PATHWAY”, “WNTBEvTACATENINSIGNALING”, “APOPTOSIS”, and “HYPOXIA”. The enrichment results of the above gene sets indicate the effect of lncRNA ZFHX4-AS1 on immune response as well as its role in cell proliferation, apoptosis, and cell cycle in OvCa.

### The Correlation Between LncRNA ZFHX4-AS1 and Tumor-Infiltrating Immune Cells in the Tumor Microenvironment

**Figure 7:**
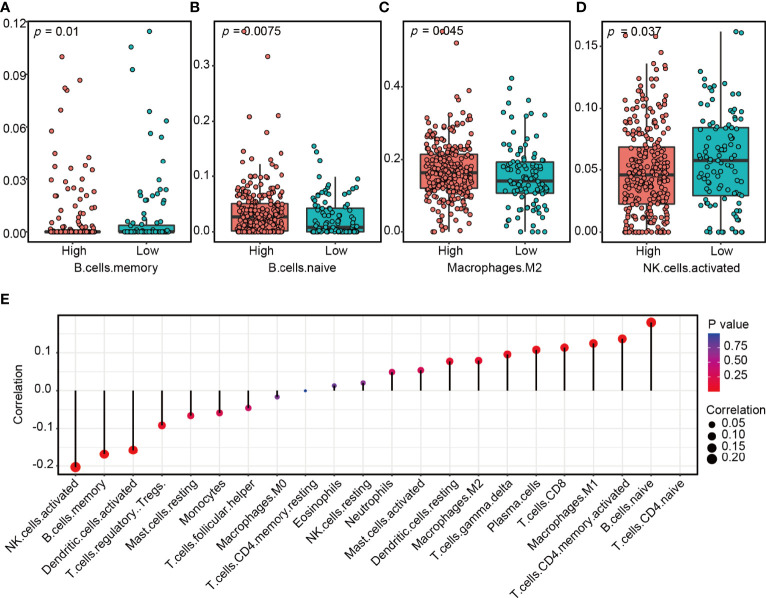
The lncRNA ZFHX4-AS1 expression affects immune cell infiltration in the tumor microenvironment of OvCa. A significant correlation exists between ZFHX4-AS1 expression and **(A)** memory B cells, **(B)** naive B cells, **(C)** M2 macrophages, **(D)** activated NK cells. **(E)** Correlation between lncRNA ZFHX4-AS1 and 22 kinds of tumor-infiltrating immune cells in OvCa.

To investigate the effects of lncRNA ZFHX4-AS1 expression on the tumor immune microenvironment, the CIBERSORT R package was used to analyze the 22 tumor-infiltrating immune cells of the tumor samples in TCGA database. As shown in **Figure 7**, there was a significant correlation between lncRNA ZFHX4-AS1 expression and different types of immune cells, including memory B cells (*P* = 0.01) (**Figure 7A**), naive B cells (*P* = 0.0075) (**Figure 7B**), M2 macrophages (*P* = 0.045) (**Figure 7C**), and activated NK cells (*P* = 0.037) (**Figure 7D**). In the GSE18520 dataset, we also found that lncRNA ZFHX4-AS1 expression was significantly correlated with 9 kinds of immune cells, including naive B cells (*P* = 0.012) and activated NK cells (*P* = 1.8e−06) (**Supplementary Figure 3**). In addition, the collected OvCa samples were divided into high and low expression groups according to the expression level of lncRNA ZFHX4-AS1. RT-qPCR results confirmed that M2 macrophage surface marker CD206 was significantly increased in the high expression group (*P* = 0.021) (**Supplementary Figure 4A**). These results suggest that lncRNA ZFHX4-AS1 plays an important role in immune cell infiltration during tumor pathology.

### The Prognostic Value of LncRNA ZFHX4-AS1 and Its Related Genes in OvCa

**Figure 8:**
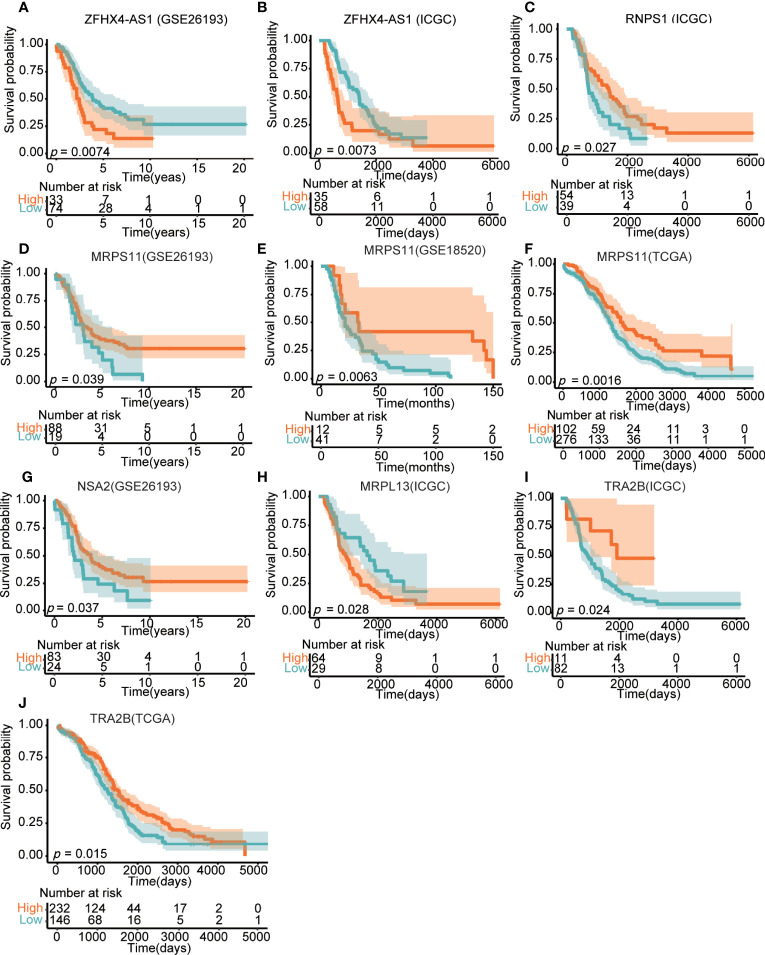
Effects of lncRNA ZFHX4-AS1 and its related DEGs on the overall survival in different databases, involving **(A)** ZFHX4-AS1 in GSE26193, **(B)** ZFHX4-AS1 in ICGC, **(C)** *RNPS1* in ICGC, **(D)** *MRPS11* in GSE26193, **(E)** *MRPS11* in GSE18520, **(F)** *MRPS11* in TCGA, **(G)** *NSA2* in GSE26193, **(H)** *MRPL13* in ICGC, **(L)** *TRA2B* in ICGC, **(J)** *TRA2B* in TCGA.

Based on the analysis in GEO and ICGC, the effect of lncRNA ZFHX4-AS1 on the OS of patients with OvCa was first demonstrated in the validation set. Kaplan–Meier survival curves revealed that the high lncRNA ZFHX4-AS1 expression group had poor OS (GSE26193:*P* = 0.0074, ICGC:*P* = 0.0073) (**Figures 8A, B**). To evaluate the impact of DEGs related to lncRNA ZFHX4-AS1on the prognosis of OvCa, the prognostic value of DEGs with top-five degrees in the TCGA, GEO, and ICGC databases was investigated. As shown in **Figures 8C–G, I, J**, the upregulation of *RNPS1* (ICGC:*P* = 0.027), *MRPS11* (TCGA:*P* = 0.0016, GSE26193:*P* = 0.039, GSE18520:*P* = 0.0063), *NSA2*(GSE26193:*P* = 0.037), and *TRA2B* (TCGA: *P* = 0.015, ICGC:*P* = 0.024) were significantly correlated with better OS in OvCa. On the contrary, *MRPL13*(ICGC:*P* = 0.028) (**Figure 8H**) mRNA upregulation signified a worse prognosis. Hence, lncRNA ZFHX4-AS1 and its related genes play important roles in assessing the prognosis of OvCa.

### Regulation of Immune Molecules by LncRNA ZFHX4-AS1 and Its Related Genes

**Figure 9:**
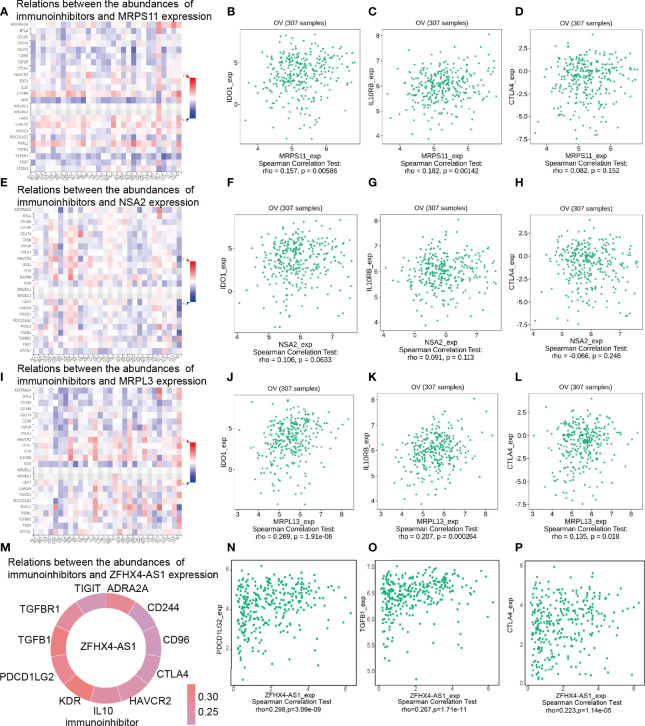
Spearman’s correlation between lncRNA ZFHX4-AS1 and its related DEGs and immunoinhibitors (TISIDB). **(A)** Relations between the *MRPS11* expression and abundances of immunoinhibitors, including **(B)** IDO1, **(C)** IL10RB, and **(D)** CTLA4 in OvCa. **(E)** Relationship between the *NSA2* expression and abundances of immunoinhibitors, including **(F)** IDO1, **(G)** IL10RB, and **(H)** CTLA4 in OvCa. **(I)** Relationship between the *MRPL13* expression and abundances of immunoinhibitors, including **(J)** IDO1, **(K)** IL10RB, and **(L)** CTLA4 in OvCa. **(M)** Relationship between the ZFHX4-AS1 expression and the abundances of immunoinhibitors, including **(N)** PDCD1LG2, **(O)** TGFB1, and **(P)** CTLA4 in OvCa.

The TISIDB database was adopted to analyze the Spearman’s correlation between the expression of key DEGs and immunoinhibitors. As shown in **Figure 9**, *MRPS11*, *NSA2*, and *MRPL13* were positively associated with immunoinhibitors IDO1 and IL10RB. Among them, *MRPS11* was significantly correlated with IDO1 (*P* = 0.00586) (**Figure 9B**) and IL10RB (*P* = 0.00142) (**Figure 9C**) and *MRPL13* was evidently linked to IDO1 (*P* = 1.91e−06) (**Figure 9J**) and IL10RB (*P* = 0.000264) (**Figure 9K**). Specifically, there was a significant association between *MRPL13* and cytotoxic T lymphocyte-associated antigen-4 (CTLA-4) (*P* = 0.018) (**Figure 9L**). Spearman correlation analysis was performed on lncRNA ZFHX4-AS1 and immune checkpoints. As shown in **Figure 9M**, lncRNA ZFHX4-AS1 was positively correlated with 11 immune suppression checkpoints (Spearman’s r > 0.2, *P* value < 0.05). PDCD1LG2 (*P* = 3.09 e−09), TGFβ1 (*P* = 1.71 e−11), and CTLA4 (*P* = 1.14 e−05) were all significantly correlated with lncRNA ZFHX4-AS1 (**Figures 9N–P**). The correlations between lncRNA ZFHX4-AS1 and immune checkpoints in GSE18520 and GSE26193 data sets were also shown in **Supplementary Figure 5**. LncRNA ZFHX4-AS1 was significantly associated with PDCD1-LG2 (*P* =0.0004 in GSE18520, *P* = 0.0024 in GSE26193) and CTLA4 (*P* < 2.2e−16 in GSE18520, *P* = 0.017 in GSE26193) in both data sets. RT-qPCR results showed that PDCD1LG2 expression was positively correlated with lncRNA ZFHX4-AS1 (*P* = 0.037) (**Supplementary Figure 4B**), while CTLA4 expression was increased but not statistically significant in lncRNA ZFHX4-AS1 high expression group (*P* = 0.1088) (**Supplementary Figure 4C**).

### The Expression of lncRNA ZFHX4-AS1 in OvCa Tissues and Cell Lines

**Figure 10:**
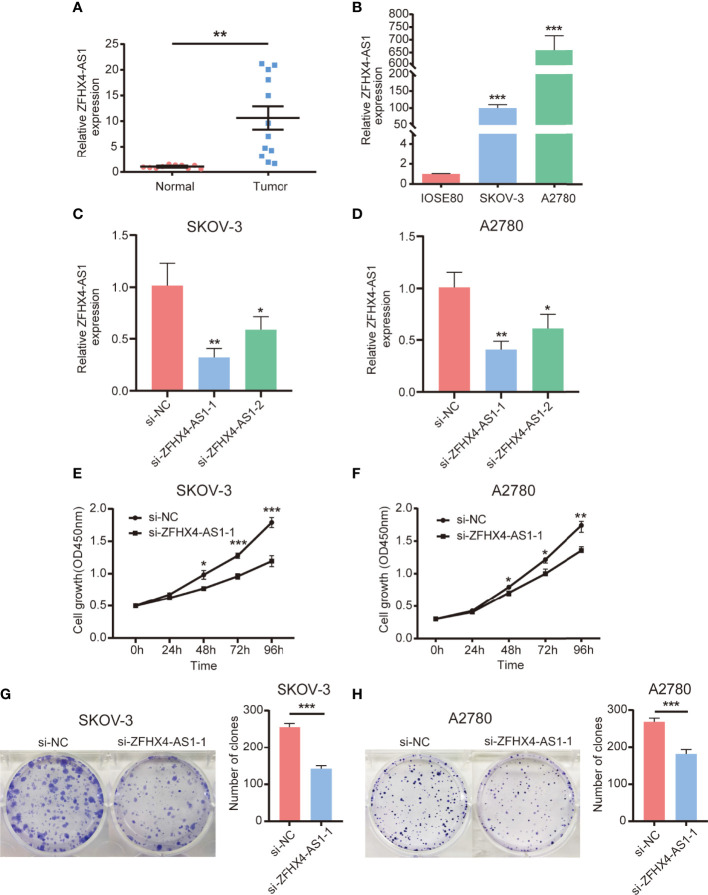
Expression of lncRNA ZFHX4-AS1 in OvCa tissues and cells, and its promoting effect on OvCa cells proliferation. **(A)** The expression of lncRNA ZFHX4AS1 in OvCa and the relative normal tissues was detected by RT-qPCR; **P = 0.001. **(B)** The expression of lncRNA ZFHX4AS1 in IOSE80, SKOV-3, and A2780 cells detected by RT-qPCR, with the normal ovarian epithelial cell line IOSE80 set as control; ***P < 0.001; *n* = 3, mean ± SD. **(C) (D)** The knockdown efficiency of si-ZFHX4-AS1-1 and si-ZFHX4-AS1-2 compared with the negative control (NC) in SKOV-3 and A2780 cells was analyzed *via* RT-qPCR; **P<0.01; *n* = 3, mean ± SD. **(E-H)** CCK-8 assays and colony formation assays of SKOV-3 and A2780 cells transfected with si-NC and si-ZFHX4-AS1-1. *P < 0.05, **P < 0.01, ***P < 0.001; *n* = 3, mean ± SD.

Based on the RT-qPCR results of cancer tissues from 12 patients and normal ovarian tissues from 10 patients, lncRNA ZFHX4-AS1 was found to be significantly highly expressed in the OvCa tissues when compared with the normal ovarian tissues (*P* = 0.001) (**Figure 10A**). Furthermore, the expression of lncRNA ZFHX4-AS1 in OvCa cell lines SKOV-3 and A2780 was tested, and the results were compared with those from the normal ovarian cell line IOSE80 using RT-qPCR. It was found that A2780 (*P* < 0.001) and SKOV-3 (*P* < 0.001) had higher lncRNA ZFHX4-AS1 expression levels than IOSE80 (**Figure 10B**).

### LncRNA ZFHX4-AS1 Is Associated With Cell Proliferation *In Vitro*

To estimate whether the expression of lncRNA ZFHX4-AS1 had the potential to influence the proliferation of OvCa cells, assays with the underexpression of lncRNA ZFHX4-AS1 were performed. The two siRNAs were created to knockdown lncRNA ZFHX4-AS1 expression, and RT-qPCR analysis was used to measure the relative lncRNA ZFHX4-AS1 expressions in SKOV-3 and A2780 cells. LncRNA ZFHX4-AS1 was more effectively knocked down by si-ZFHX4-AS1-1 in SKOV-3 (*P* < 0.01) (**Figure 10C**) and A2780 (*P* < 0.01) (**Figure 10D**). CCK-8 assays and colony formation assays showed the suppression of lncRNA ZFHX4-AS1 significantly inhibited cell proliferation and colony-formation abilities in SKOV-3 (**Figures 10E, G**) and A2780 (**Figures 10F, H**).

### LncRNA ZFHX4-AS1 Promotes Cell Invasion, Migration and EMT in OvCa

**Figure 11:**
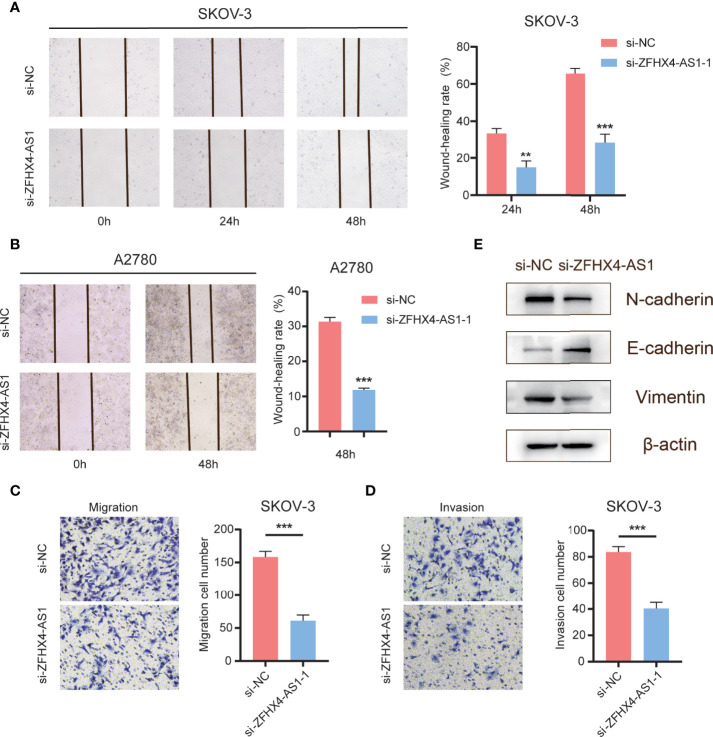
The effects of lncRNA ZFHX4-AS1 promoting OvCa cells migration, invasion and EMT. **(A) (B)** Wound healing assays of SKOV-3 and A2780 cells transfected with si-NC and si-ZFHX4-AS1-1. **P < 0.01, ***P < 0.001; n = 3, mean ± SD. **(C) (D)** Transwell migration and invasion assays of SKOV-3 cells transfected with si-NC and si-ZFHX4-AS1-1. ***P < 0.001; n = 3, mean ± SD. **(E)** WB of EMT-related markers in si-ZFHX4-AS1-1 and si-NC groups.

We verified the effect of lncRNA ZFHX4-AS1 on cell metastasis in OvCa. As shown in **Figures 11A, B**, the knockdown of lncRNA ZFHX4-AS1 inhibited the migration of SKOV-3 and A2780 cells in the wound healing experiment. Furthermore, transwell assays proved that the migration and invasion ability of SKOV-3 cells in si-ZFHX4-AS1-1 group were decreased compared with si-NC group (**Figures 11C, D**). In addition, the effect of lncRNA ZFHX4-AS1 on EMT-related markers was also confirmed by western blotting. As shown in **Figure 11E**, the expression of the epithelial marker E-cadherin was higher in the si-ZFHX4-AS1-1 group, whereas the expression of mesenchymal markers (N-cadherin, Vimentin) was increased in the si-NC group.

## Discussion

OvCa has always been the primary cause of death in gynecological malignancies. Lack of early diagnostic biomarkers is one of the major reasons for the delayed diagnosis and poor prognosis of patients with OvCa (15).As an oncogene or tumor suppressor gene, lncRNA could serve as a potential diagnostic and prognostic biomarker and therapeutic target in several cancers, including OvCa (16, 17).Based on the database search, lncRNA ZFHX4-AS1 was perceived to be highly expressed in OvCa. Although it has been verified that lncRNA ZFHX4-AS1 is upregulated in bladder cancer and acts as an oncogene in breast cancer (13, 14), its role in OvCa has not been established.

In our study, bioinformatics analysis using TCGA and GTEx high-throughput RNA sequencing data suggested that lncRNA ZFHX4-AS1 expression was significantly elevated in OvCa when compared with normal tissues. The high expression of lncRNA ZFHX4-AS1 was associated with advanced clinicopathological features such as higher FIGO stage and older age, and negatively impacted OS and PFS. In the GEO and ICGC cohorts, lncRNA ZFHX4-AS1 was also found to be significantly correlated with the poor outcomes of patients with OvCa. In our multivariate Cox regression model, lncRNA ZFHX4-AS1 expression was an independent prognostic factor in OvCa. As shown in the calibration graphs, a good consistency was noted between the observed and predicted levels of OS and PFS at 1, 2, and 3 years. Furthermore, the high expression of lncRNA ZFHX4-AS1 was verified in the OvCa tissues and cell lines, and its promoting effect on proliferation, clonal formation, invasion and migration was confirmed through vitro experiments. As per the findings, it could be stated that lncRNA ZFHX4-AS1 was upregulated in many patients with OvCa, and its potential as a diagnostic and prognostic biomarker warrants further experimental and clinical validation.

In the functional analysis of lncRNA ZFHX4-AS1, it was found to be related to the composition of mitochondria, aerobic respiration, and cell metabolism. Mitochondria are the energy factories in the cells, and cancer cells are no exception. Studies have confirmed that if cancer cells are genetically engineered to prevent them from breathing, they cannot proliferate without other interventions (18). The analysis of the lncRNA ZFHX4-AS1 related mechanism revealed the presence of several classic cancer-related genes or signaling pathways, such as “MYCTARGETSV1”, “P53PATHWAY” and “WNTBETACATENINSIGNALING”, which signifies its involvement in tumor genesis and development (19–21). Moreover, lncRNA ZFHX4-AS1 is associated with epithelial–mesenchymal transformation (EMT) and hypoxia. We also verified the role of lncRNA ZFHX4-AS1 in promoting EMT through western blotting. EMT is a cellular procedure that confers cancer cells with increased tumor-initiation and metastasis potential as well as greater resistance to elimination by several treatment regimens (22). Hypoxia creates intra-tumor oxygen gradients that contribute to tumor plasticity and heterogeneity and promote highly aggressive and metastatic phenotypes (23). Recent studies have indicated that hypoxia is associated with poor prognosis in patients by regulating the tumor microenvironment (24).

In our study, lncRNA ZFHX4-AS1 was observed to participate in the regulation of multiple inflammatory signaling pathways in OvCa, such as IL2/STAT5 and TNF-α/NF-κB signaling pathways, which suggests its role in the creation of a suppressive immune microenvironment. Recent studies have proven that IL2/STAT5 signaling pathway induces depletion of CD8+T cells in the tumor microenvironment, thereby inhibiting anti-tumor immunity and promoting immune escape of the tumor cells (25). Lim et al. found that TNF-α/NF-κB signaling pathway is a major factor that triggers cancer-cell immunosuppression to resist T cell surveillance by stabilizing programmed cell death ligand 1 (PD-L1) (26).

To further examine the relationship between lncRNA ZFHX4-AS1 and immune infiltration levels in OvCa, CIBERSORT R package was used for the statistical analysis. A significant correlation was perceived between lncRNA ZFHX4-AS1 expression and the immune infiltration levels of M2 macrophages, activated NK cells, memory B cells, and naïve B cells. In the lncRNA ZFHX4-AS1 high expression group, M2 macrophage infiltration level was higher in OvCa than in the normal cells. M2 repair-type macrophages are widely known to stimulate tumor growth by releasing growth-promoting molecules (27). In many types of malignancies, such as colorectal cancer, gastric cancer, breast cancer, and lung cancer, M2-polarized tumor-associated macrophages play a key role in cancer progression and metastasis. Moreover, the mechanism affecting M2-polarized macrophages has been further researched (28–31). Previous studies have identified lncRNA-MM2P to be a regulator of macrophage M2 polarization and have shed light on its role in macrophage tumorigenesis (32). In our study, the surface marker CD206 of M2 macrophages was detected to be enriched in the high expression group of lncRNA ZFHX4-AS1 by RT-qPCR, providing possible evidence that lncRNA ZFHX4-AS1 affects the infiltration of M2 macrophages in OvCa. NK cells have the potential to kill tumor cells in various ways without prior sensitization (33).This unique property of the immune cells and their ability to enhance T cell responses and antibody support the role of NK cells as anticancer agents (34).In our study, NK cells were significantly enriched in the low expression group of lncRNA ZFHX4-AS1. Based on these findings, it could be hypothesized that lncRNA ZFHX4-AS1 might lead to poor prognosis by influencing the infiltrating landscape of immune cells in OvCa.

At present, tumor immunotherapy has gained prominence in cancer treatment, and the research hotspots are focused on immune checkpoint inhibitors. Immune checkpoint inhibitors have shown extraordinary potential in several areas, including the targeting of CTLA-4 and programmed cell death protein 1 (PD-1) with its ligand PD-L1 pathway (35). Our results reveal that lncRNA ZFHX4-AS1 is significantly related to 11 immunoinhibitors, and verified the enhanced expression of PDCD1LD2 and CTLA4 when lncRNA ZFHX4-AS1 was highly expressed in OvCa. In earlier 2011, the US Food and Drug Administration approved anti-CTLA-4 for the treatment of advanced stage melanoma (36). Immunotherapy targeting PD1/PD-L1 has been approved as an adjunctive therapy for different cancers with considerable success in the past few years. PD-L2 is the second ligand of PD-1 and shows overlapping function with PD-L1 (37). Several studies have demonstrated that PD-L2 is associated with patient outcomes in different cancers (38).Neutralization of PD-L2 has been shown to be crucial for overcoming immune checkpoint resistance in OvCa (39). The effect of lncRNA ZFHX4-AS1 on the expression of PDCD1LG2 and CTLA4 in OvCa suggests that it may be a predictive biomarker of immunotherapy response in OvCa patients. TGF-β is a key negative immunomodulator of immune balance, which inhibits various targets in the immune system and causes tumor immune escape and tumor immunotherapy adverse reactions (40, 41). There is a report that blocking TGF-β along with immune checkpoint therapy increases Th1 subsets and promotes clonal expansion of CD8 T cells, thereby leading to the regression of bone castration-resistant prostate cancer and improved survival (42). In addition, the genes *MRPS11* and *MRPL13*, which are related to lncRNA ZFHX4-AS1, have been found to be significantly associated with indoleamine 2, 3-dioxygenase 1 (IDO1). IDO1 is overexpressed in different malignancies and is associated with poor prognosis (43). IDO1 is an enzyme that plays a role in the metabolism of the essential amino acid l-tryptophan to l-kynurenine. The mechanisms by which IDO1 and kynurenine metabolites promote tumorigenesis include the shaping of a tumor-friendly immune microenvironment and the activation of aryl hydrocarbon receptor (44). IDO1, in combination with other immunotherapeutics such as PD-1/PD-L1 or CTLA-4 inhibitors, has an objective response rate that varies between 10% and 57% in different cancer types (45, 46). It has been demonstrated that IDO1 inhibition can enhance the efficacy of radiotherapy in colorectal cancer (47). To date, clinical trials on precision medicine therapy targeting IDO1 are ongoing.

Although the findings of this study are helpful in deciphering the relationship between lncRNA ZFHX4-AS1 and OvCa, the research on its role in cancer is still in its infancy. Owing to the differences among the databases, limited sample size, and few relevant experimental studies, the functional mechanism of lncRNA ZFHX4-AS1 needs further experimental verification. However, this research has several advantages too. This is the first time that lncRNA ZFHX4-AS1 has been found to be associated with proliferation and metastasis of OvCa cells and multiple key cancer-related pathways and immunosuppressive signaling pathways. This study is also the first one to investigate the effect of lncRNA ZFHX4-AS1 on immune cell infiltration in the tumor immune microenvironment. Furthermore, the immune checkpoints related to lncRNA ZFHX4 were explored. These findings provide a research direction for understanding the role of lncRNA ZFHX4-AS1 in immunotherapy for OvCa. We intend to continue exploring the functional mechanism by which lncRNA ZFHX4-AS1 promotes the progression of OvCa.

## Conclusions

In summary, our research has shown that lncRNA ZFHX4-AS1 is significantly highly expressed in OvCa samples and that its high expression is associated with poor outcomes. In terms of biological function, lncRNA ZFHX4-AS1 is closely related to tumor occurrence and developments well as the establishment of an adverse tumor immune microenvironment. Investigation of lncRNA ZFHX4-AS1 and its related genes correlated with immune checkpoints has enhanced our understanding of the low response of OvCa to immunotherapy.

## Data Availability Statement

The datasets presented in this study can be found in online repositories. The names of the repository/repositories and accession number(s) can be found in the article/**Supplementary Material**.

## Ethics Statement

The studies involving human participants were reviewed and approved by The Ethics Committee of Harbin Medical University Cancer Hospital. The patients/participants provided their written informed consent to participate in this study.

## Author Contributions

GL: guidance and supervision. XW and YW: investigation, analysis and writing the manuscript. FS and ZZ: collection of clinical samples. YX, CY and LZ: visualization and editing. GL: Final review and authorization. All authors contributed to this research and presented the final achievement.

## Funding

This study was supported by the National Natural Science Foundation of China (No. 81872507), Harbin Medical University Cancer Hospital (CN) Nn10 Project (Nn10py2017-01), key projects of Haiyan Fund (JJZD2017-01), and key projects of Heilongjiang Natural Science Foundation(ZD2020H007), the Heilongjiang Province Doctoral Post-doctoral Fund (LBH-Z21177), the Harbin Medical University Haiyan Youth Fund (JJQN2022-3), the Fundamental Research Funds for the Provincial Universities.

## Conflict of Interest

The authors state that the research was conducted without any commercial or financial relationships that could be interpreted as potential conflicts of interest.

## Publisher’s Note

All claims expressed in this article are solely those of the authors and do not necessarily represent those of their affiliated organizations, or those of the publisher, the editors and the reviewers. Any product that may be evaluated in this article, or claim that may be made by its manufacturer, is not guaranteed or endorsed by the publisher.
