# Supplementary figures and images for: Novel LncRNA ZFHX4-AS1 as a Potential Prognostic Biomarker That Affects the Immune Microenvironment in Ovarian Cancer
Source: Front Oncol. 2022 Jul 12;12:945518. doi: 10.3389/fonc.2022.945518 (PMC9315108; doi:10.3389/fonc.2022.945518)

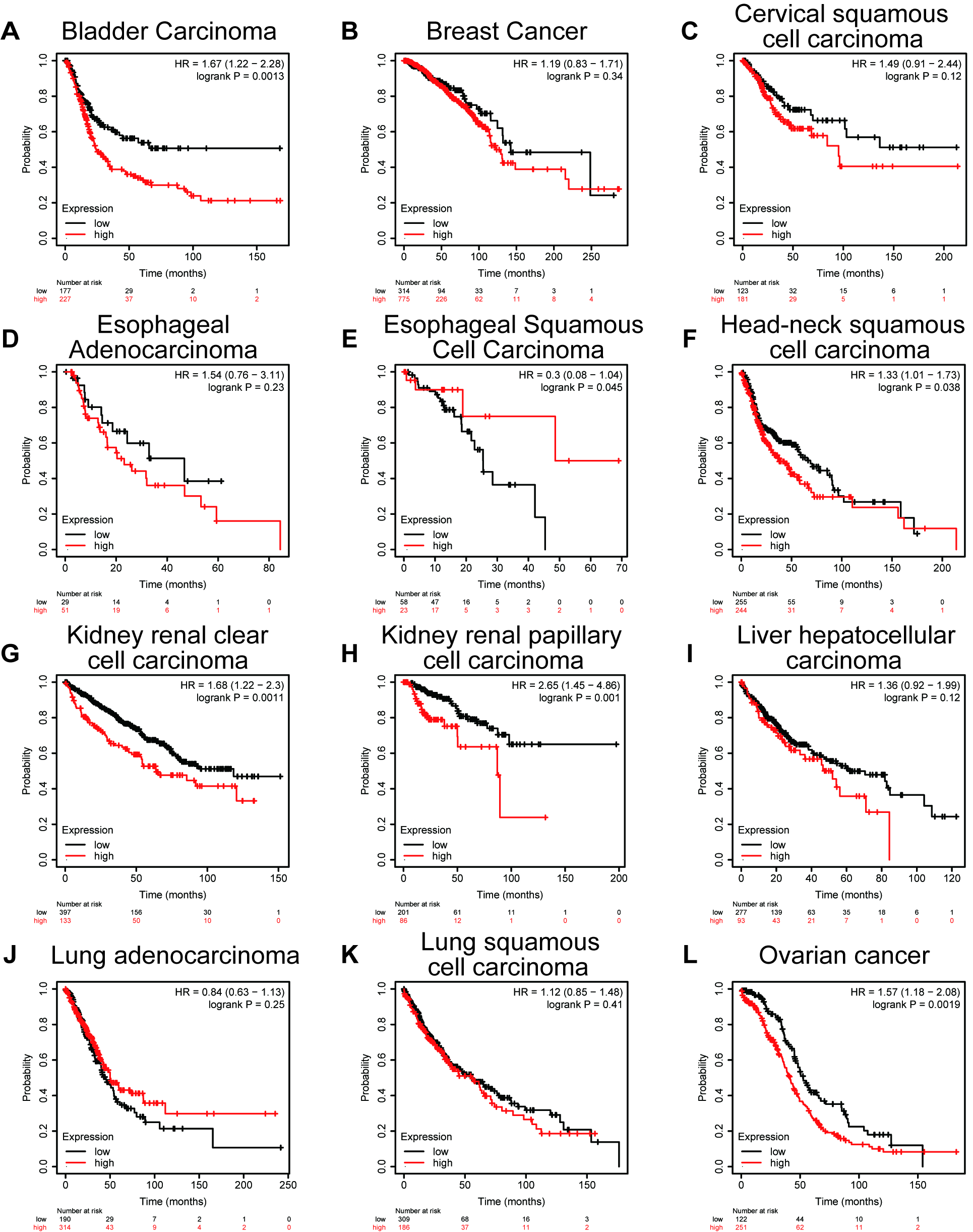

Supplement: Supplementary file 1 [file Image_1.tif]

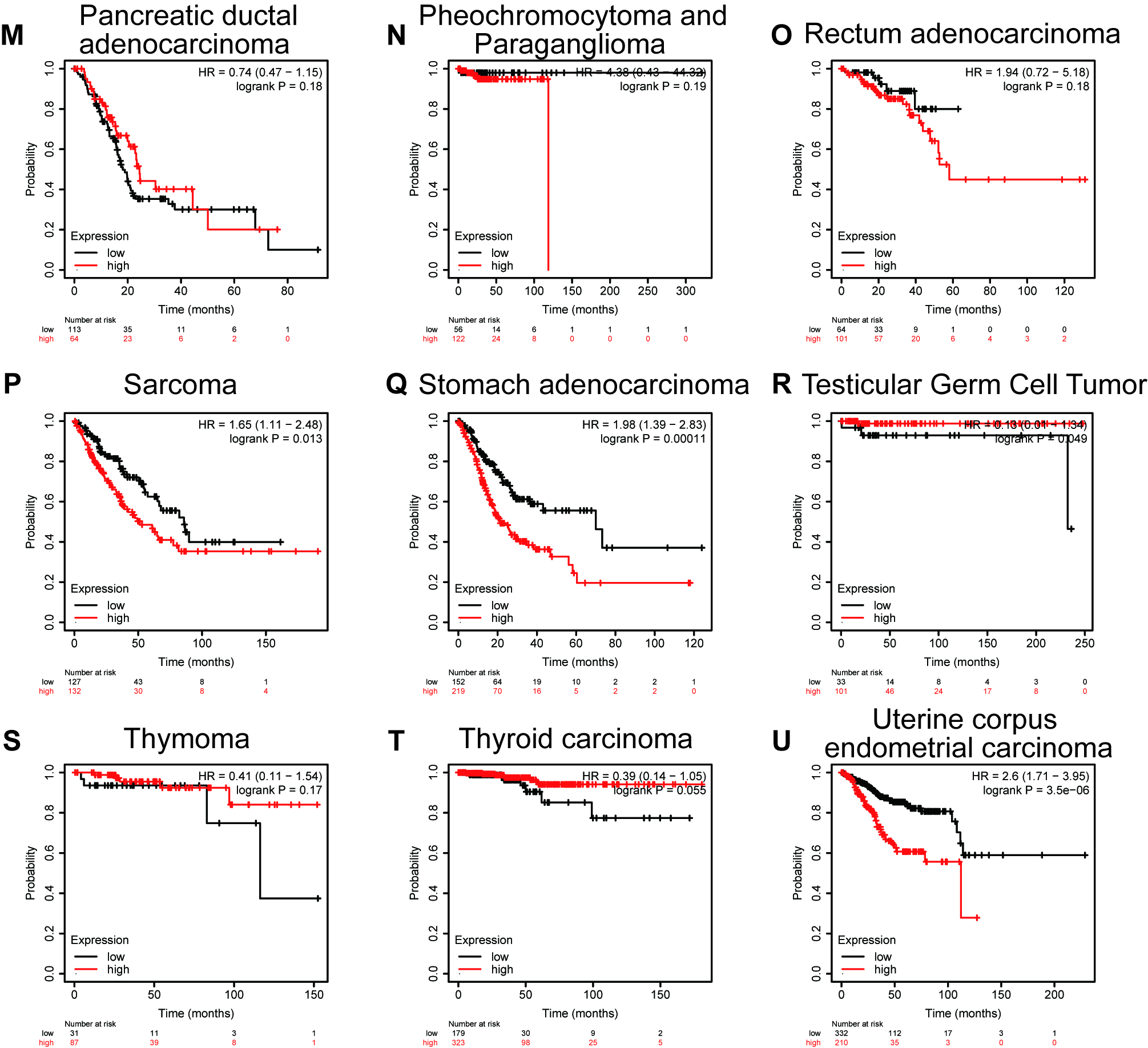

Supplement: Supplementary file 2 [file Image_2.tif]

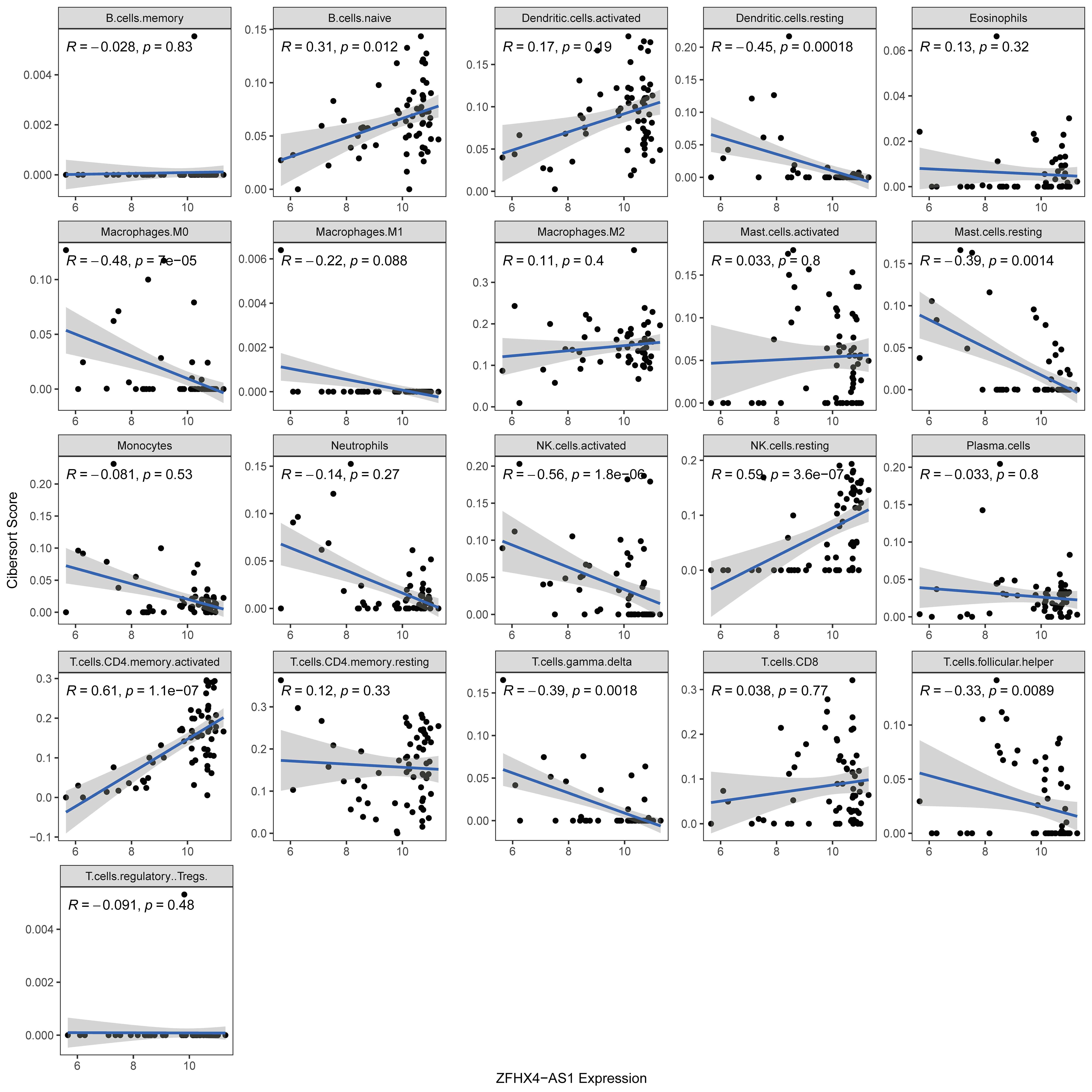

Supplement: Supplementary file 3 [file Image_3.tiff]

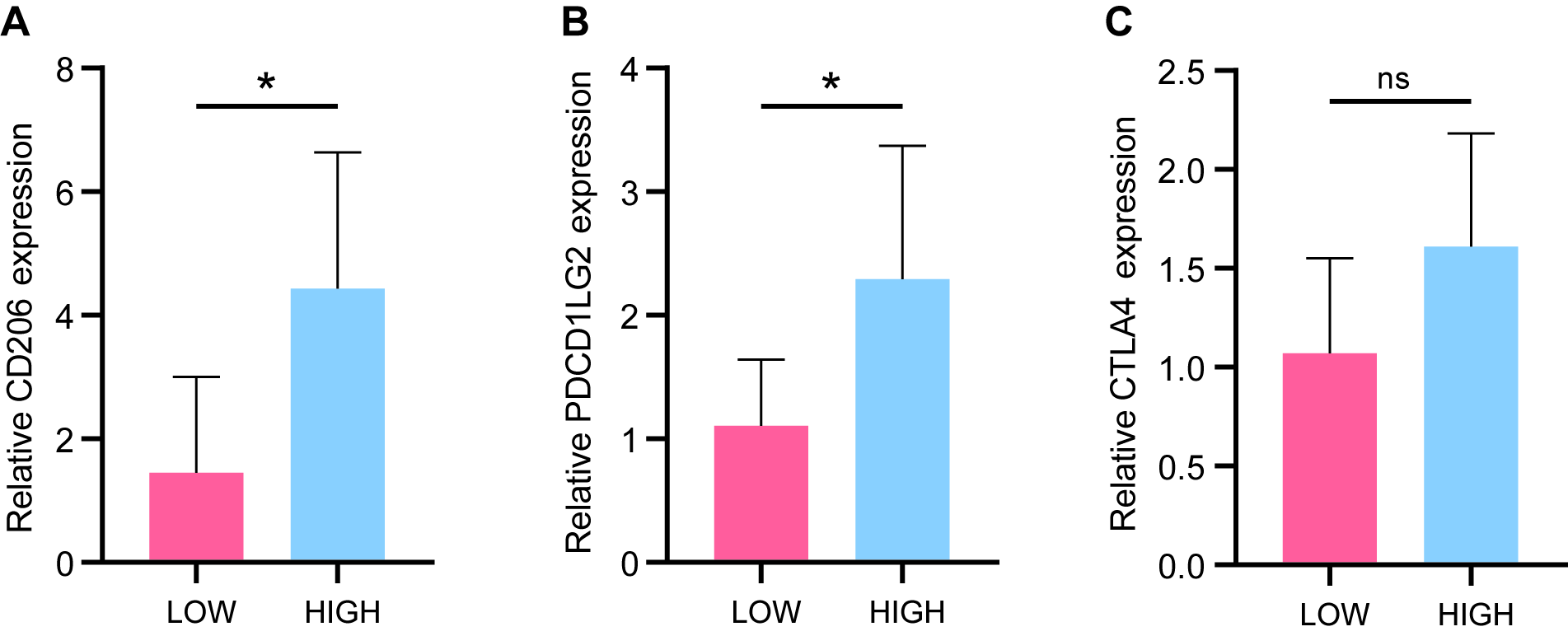

Supplement: Supplementary file 4 [file Image_4.tif]

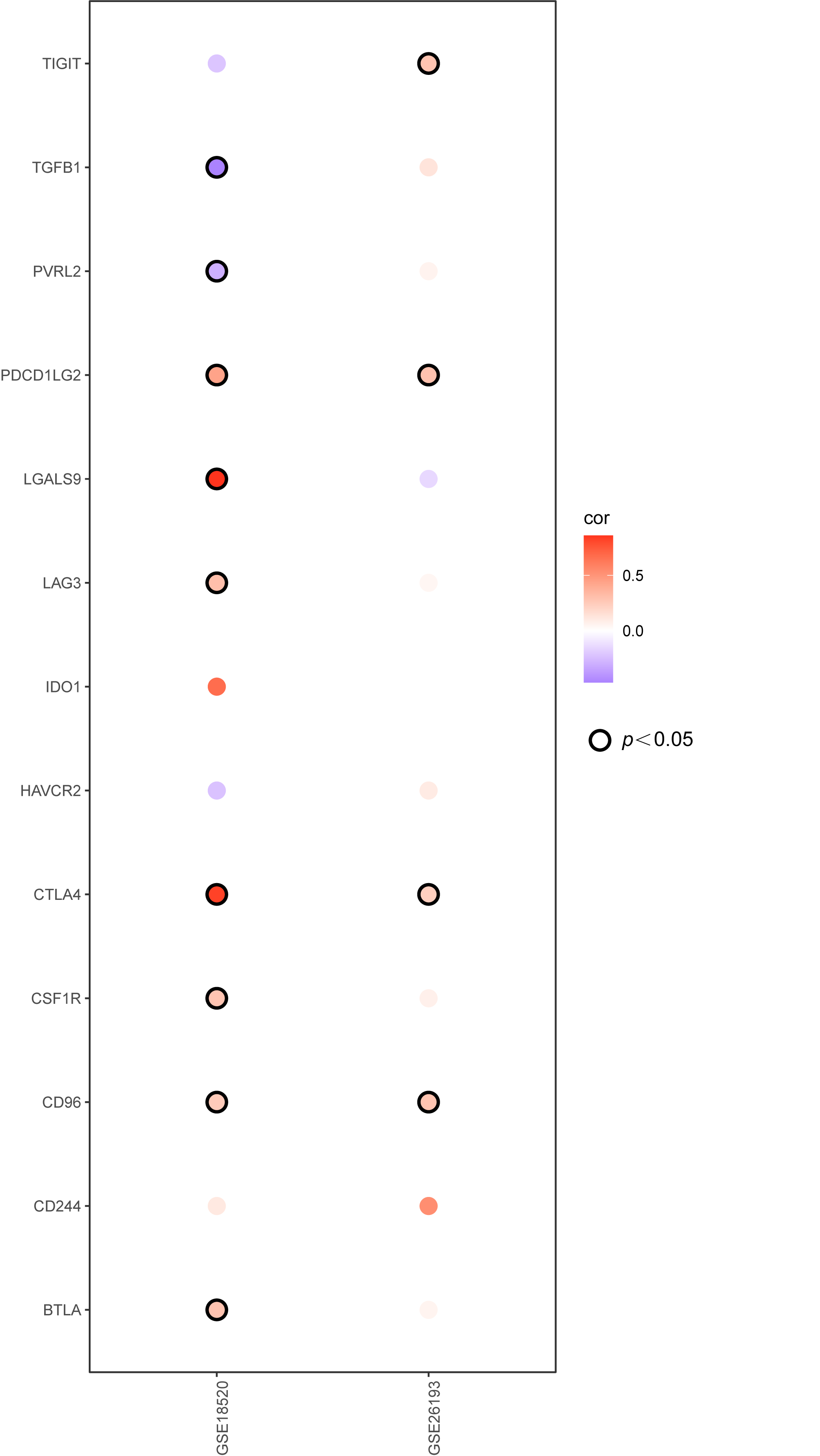

Supplement: Supplementary file 5 [file Image_5.tif]
