# Supplementary material for: Novel LncRNA ZFHX4-AS1 as a Potential Prognostic Biomarker That Affects the Immune Microenvironment in Ovarian Cancer
Source: Front Oncol. 2022 Jul 12;12:945518. doi: 10.3389/fonc.2022.945518 (PMC9315108; doi:10.3389/fonc.2022.945518)
Supplement: Supplementary file 6 [file Table_1.docx]

**Supplementary Table 1.** Clinicopathologic characteristics of OvCa tissues

| Characters | Level | Sample |
| --- | --- | --- |
| Stage | Stage I | 8 |
|  | Stage II | 2 |
|  | Stage III+IV | 2 |
| Grade | Grade1+2 | 1 |
|  | Grade3 | 11 |
| Histological | Serous adenocarcinoma | 9 |
|  | Mucoid adenocarcinoma | 1 |
|  | Endometrioid adenocarcinoma | 1 |
|  | Clear cell carcinoma | 1 |
| Age | <60 | 6 |
|  | >=60 | 6 |
| CA125 | High | 11 |
|  | Normal | 1 |
| Comorbidities | Yes | 0 |
|  | No | 12 |
| Accompanied malignancies | Yes  No | 0  12 |
