# Supplementary material for: Novel LncRNA ZFHX4-AS1 as a Potential Prognostic Biomarker That Affects the Immune Microenvironment in Ovarian Cancer
Source: Front Oncol. 2022 Jul 12;12:945518. doi: 10.3389/fonc.2022.945518 (PMC9315108; doi:10.3389/fonc.2022.945518)
Supplement: Supplementary file 7 [file Table_2.docx]

**Supplementary Table 2. The primers for qRT-PCR in the current study**

| **Gene** | **Primers (5’ to 3’)** |
| --- | --- |
| GAPDH  ZFHX4-AS1  PDCD1LG2  CD206  CTLA4 | Forward: ATGGAGAAGGCTGGGGCTC  Reverse: AAGTTGTCATGGATGACCTTG  Forward: CTGCCTCTTCCCGCTTTAAT  Reverse: GCCAGCCGGTGATTTGATA  Forward: ATCCAACTTGGCTGCTTCAC  Reverse: CTCCCAAGACCACAGGTTCA  Forward: GGGTTGCTATCACTCTCTATGC  Reverse: TTTCTTGTCTGTTGCCGTAGTT  Forward: TTTCTTCTCTTCATCCCTGTCTTCTGC  Reverse: TAAATCTGGGTTCCGTTGCCTATGC |
